# Pain perception and morbidity after peri-implant surgeries in patients with and without temporomandibular disorder - A clinical study

**DOI:** 10.4317/jced.62332

**Published:** 2024-12-01

**Authors:** Mateus Fiuza Santos, Yago Warles Silva Pereira, Luiz Renato Paranhos, Antônio Sérgio Guimarães, João Antônio Chaves de Souza, Rogério Heládio Lopes Motta, Juliana Cama Ramacciato

**Affiliations:** 1 MSc student, Postgraduate Program in Pain and Temporomandibular Disorders, São Leopoldo Mandic College, São Paulo, Brazil; 2 MSc student, Postgraduate program in Dentistry, Faculty of Dentistry, Federal University of Uberlândia, Minas Gerais, Brazil; 3 Department of Preventive and Community Dentistry, Faculty of Dentistry, Federal University of Uberlândia, Minas Gerais, Brazil; 4 Experimental Pain Laboratory, São Leopoldo Mandic College, Campinas, São Paulo, Brazil; 5 epartment of Periodontology, Faculty of Dentistry, Federal University of Goiás, Goiânia, Brazil; 6 Department of Pharmacology, Anesthesiology, and Therapeutics, São Leopoldo Mandic College, Campinas, São Paulo, Brazil; 7 Division of Pharmacology, Anesthesiology, and Therapeutics, São Leopoldo Mandic College, Campinas, São Paulo, Brazil

## Abstract

**Background:**

Temporomandibular disorder (TMD) involves various conditions affecting the anatomy and functional characteristics of the temporomandibular joint (TMJ). Hence, this study evaluated pain perception and morbidity after dental implant surgeries in patients with and without TMD.

**Material and Methods:**

It is a prospective randomized clinical trial with 50 participants with and without TMD, randomly selected for rehabilitation procedures with dental implants. Pain scores were recorded at seven, 14, and 21 postoperative days using a visual analog scale (VAS) for reporting pain on a scale from 0 to 10. The data were described from absolute and relative frequencies and median pain scores and then stratified between patients with and without TMD. Fisher’s exact tests compared the distribution of sex, surgery duration, and limitations for patients with and without TMD. Kruskal-Wallis tests related pain scores between the groups in the three evaluated periods. All tests applied a 5% significance level.

**Results:**

All patients (with and without TMD) subjected to peri-implant surgeries were analyzed. Pain and morbidity levels in the seventh (*p*< 0.001) and 14th (*p* = 0.002) postoperative days were higher in TMD patients. The perceived pain score in the seventh and 14th postoperative days was higher in male TMD patients than in females.

**Conclusions:**

Patients diagnosed with TMD presented higher pain and morbidity levels on the first 14 postoperative days. However, pain significantly reduced over time.

** Key words:**Temporomandibular disorder, pain, dental implant, oral rehabilitation.

## Introduction

Temporomandibular disorder (TMD) involves several conditions affecting the anatomy and functional characteristics of the temporomandibular joint (TMJ) (1). It is prevalent in about 31% of adults and 11% of children/adolescents (2), and it has a multifactorial etiology, which causes the association of biological, psychological, and social factors interacting with contextual and environmental stressors to produce painful symptomatology (3).

The most frequent TMD symptoms are facial pain, headache, limited mandibular movement, earache, neck/shoulder pain, chewing difficulty (4), and dizziness (5). The most evident signs are TMJ noise during movement, mandibular opening restriction or deviation, masseteric hypertrophy, chewing muscle tension, and bruxism/tightening (4). Both bruxism types - sleep and awake – due to TMD were related to joint pain and intra-articular disorders (6).

TMD diagnosis in the 90s relied on the Research Diagnostic Criteria for Temporomandibular Disorders (RDC/TMD) (7). More recently, the Diagnostic Criteria for Temporomandibular Disorders (DC/TMD) was created as a standardized clinical diagnosis method using two axes: I) Physical examination of masticatory structures and II) Tracking of psychosocial factors (8).

Considering the multifactorial etiology of this disorder, its treatment is also comprehensive, combining different therapeutic approaches to improve TMD treatment outcomes (9). Possible treatment alternatives are pharmacotherapy procedures, self-care techniques, intra-oral devices, muscle physical therapy, acupuncture, synovial fluid drainage, and injection for myalgia due to TMD. These alternatives are reversible and less invasive. Conversely, irreversible and more invasive procedures comprise TMJ surgical treatment and occlusal and orthodontic adjustments (4).

The literature remains limited on the relationship between oral rehabilitation with dental implants and TMD, and no studies have adequately assessed a significant association as a premise for good clinical practice (10). However, Brazão-Silva *et al*. (10) indicate two current perspectives that tend to link TMD to oral implant rehabilitation, requiring further studies for this purpose: I) A short-term risk factor related to prolonged/excessive mouth opening during surgery and sudden orthopedic changes; II) A medium- and long-term benefit, especially involving full-arch cases, such as improved patient self-perception, masticatory function comfort and stability, higher mandibular mobility, and pain reduction. Hence, this study evaluated pain perception and morbidity after dental implant surgeries in patients with and without TMD.

## Material and Methods

- Type of study and ethical criteria

It is a prospective randomized clinical trial that received previous approval from the local Ethics Committee under report #6.600.106. All participants signed an Informed Consent Form before starting the research. The privacy rights of all participants were respected, and no individual identification information was used.

- Sample selection

The study sample was selected by convenience from March to July 2024. All 50 patients were randomly allocated (www.random.org) and subjected to the DC/TMD test (11) by a single examiner (MFS) experienced and calibrated to diagnose TMD.

All 50 patients were included, provided the need for peri-implant surgical rehabilitation procedures, such as dental extractions, bone/gingival grafts, alveolar ridge regularization, single implants, and maxillary or mandibular protocol surgery. The exclusion criteria comprised the non-requirement of dental implant rehabilitation procedures, incomplete filling of the consent form and pain assessment questionnaires, and the patient’s absence in follow-up visits during the research.

- Surgical methodology

The research was conducted in the Implantology Specialization Course at the Federal University of Goiás (UFG, Goiânia, Goiás, Brazil), thoroughly following the entire aseptic chain and basic surgical technique principles. Cone-beam computed tomography (CBCT) was performed as a complementary imaging exam on each participant before surgery. The applied anesthetic technique involved 2% lidocaine anesthetic with epinephrine (1:100.000) and varied according to the area of interest for implant rehabilitation. The surgery was timed from the moment of incision to the completion of the suture and hemostatic control.

Irrigation was abundant with 0.9% sodium chloride, the suture used a 4.0 Nylon wire, and hemostasis applied sterile gauze compression. All patients were informed about postoperative care, had a follow-up visit scheduled for seven, 14, and 21 days after the procedure, and learned to fill out the visual analog scale (VAS) pain questionnaire.

Medications were only prescribed postoperatively and orally, depending on each patient’s surgery morbidity. They included dipyrone (1 g) every six hours for three to five days, paracetamol + codeine (500 mg + 30 mg) every eight hours for three days, dexamethasone (4 mg) every 12 hours for three days, and amoxicillin + potassium clavulanate (875 mg + 125 mg) every 12 hours for ten days. The suture was removed between seven and 14 days after surgery.

- Pain measurement

The patients learned to use the VAS from 0 (no pain) to 10 (maximum possible pain) and record their pain scores once a day on the seventh, 14th, and 21st days. A single examiner (MFS) provided all instructions for questionnaire filling and data collection.

The annotations were inserted in a Microsoft Excel™ 2010 (Microsoft ™ Ltd., Washington, USA) spreadsheet that also contained the patient’s registration number, age, sex, and relevant information on surgery date and duration.

- Data analysis

The data were described from absolute and relative frequencies according to sex (female or male), surgery duration (less than two hours or two hours or more), and mouth opening limitation (yes or no) on the seventh, 14th, and 21st postoperative days. The pain score (0-10) was presented from the median and interquartile range. Absolute and relative frequencies and median pain scores were stratified between patients with and without TMD. Fisher’s exact tests compared the distribution of sex, surgery duration, and limitations for patients with and without TMD. Kruskal-Wallis tests related pain scores between the groups at seven, 14, and 21 days.

Wilcoxon signed-rank tests for paired measures compared the median scores on the seventh and 14th days and the 14th and 21st days to understand pain score behavior over time.

The marginal median pain score at days seven and 14 was estimated for different sexes and patient groups with and without TMD. Hence, quantile regressions were fitted considering the median pain score as the outcome and an interaction term between sex and the occurrence of TMD as predictors. After regression adjustments, the marginal median score for the four groups (female/with TMD, female/without TMD, male/with TMD, and male/without TMD) was predicted with respective 95% confidence intervals.

Finally, focusing only on the group of TMD patients, the median pain score was compared according to sex and surgery duration at seven, 14, and 21 postoperative days using Kruskal-Wallis tests and over time with Wilcoxon signed-rank tests for paired measures. All analyses used Stata 17.0 software (StataCorp LLC, College Station, TX, USA) at a 5% significance level.

## Results

The mean age of patients included in the study was 55.2 years. Six (12%) of the 50 individuals were diagnosed with TMD before the procedure and maintained pain symptoms postoperatively ( Table 1).

**Figure 1:**
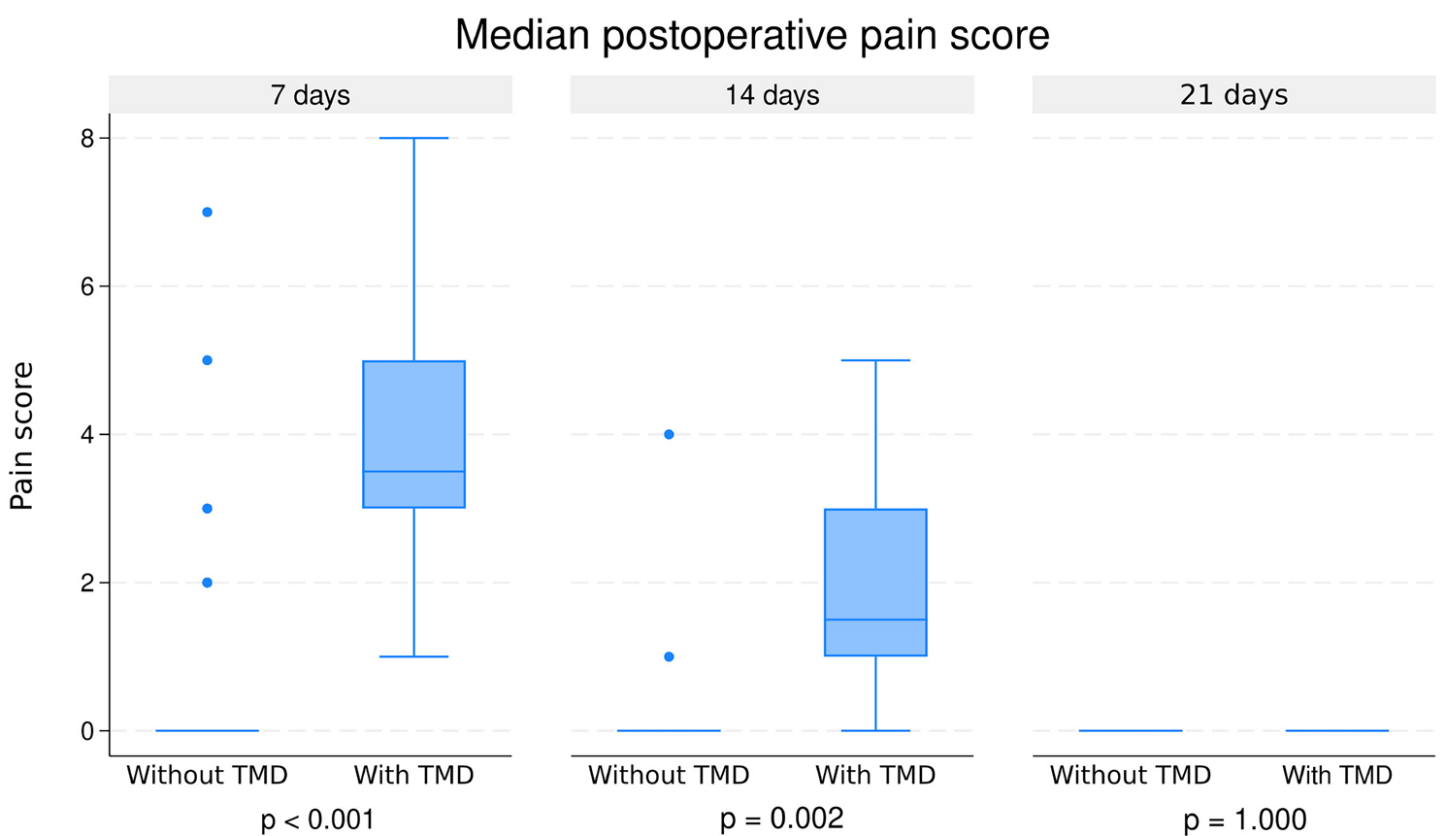
Median pain score at seven, 14, and 21 postoperative days for patients with and without TMD.

Figure 1 shows pain through the VAS seven, 14, and 21 days after surgery compared to median pain scores between patients with and without TMD.

Pain perception was higher in TMD patients at seven (*p* < 0.001) and 14 (*p* = 0.002) postoperative days. There were no statistically significant differences (*p* = 1.000) between groups after 21 days when the median pain score in both groups was zero.

**Figure 2:**
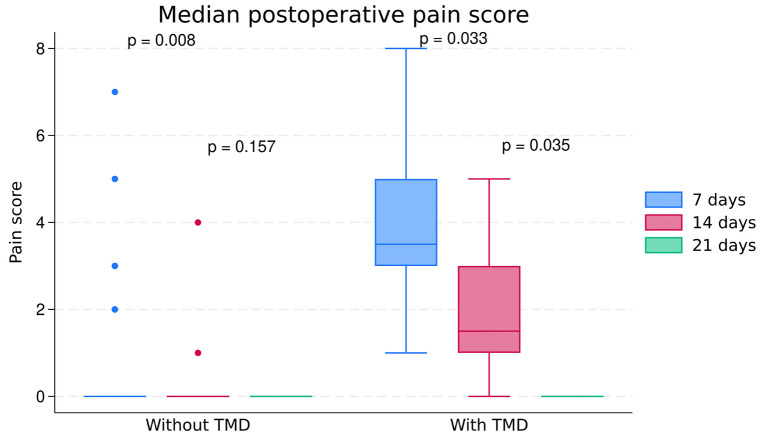
Median pain score at seven, 14, and 21 postoperative days for patients with and without TMD.

Pain perception in the group with TMD was higher at seven than 14 postoperative days (*p* = 0.033). Similarly, pain was higher at 14 than 21 postoperative days (*p* = 0.035). The median pain scores in TMD patients were 3.5, 1.5, and zero at seven, 14, and 21 postoperative days, respectively (Fig. 2). Although the group without TMD showed a median pain score of zero at seven, 14, and 21 days, some outlier values on day seven (e.g., pain scores higher than four) caused statistically significant differences in perceived pain median scores compared to day 14.

**Figure 3:**
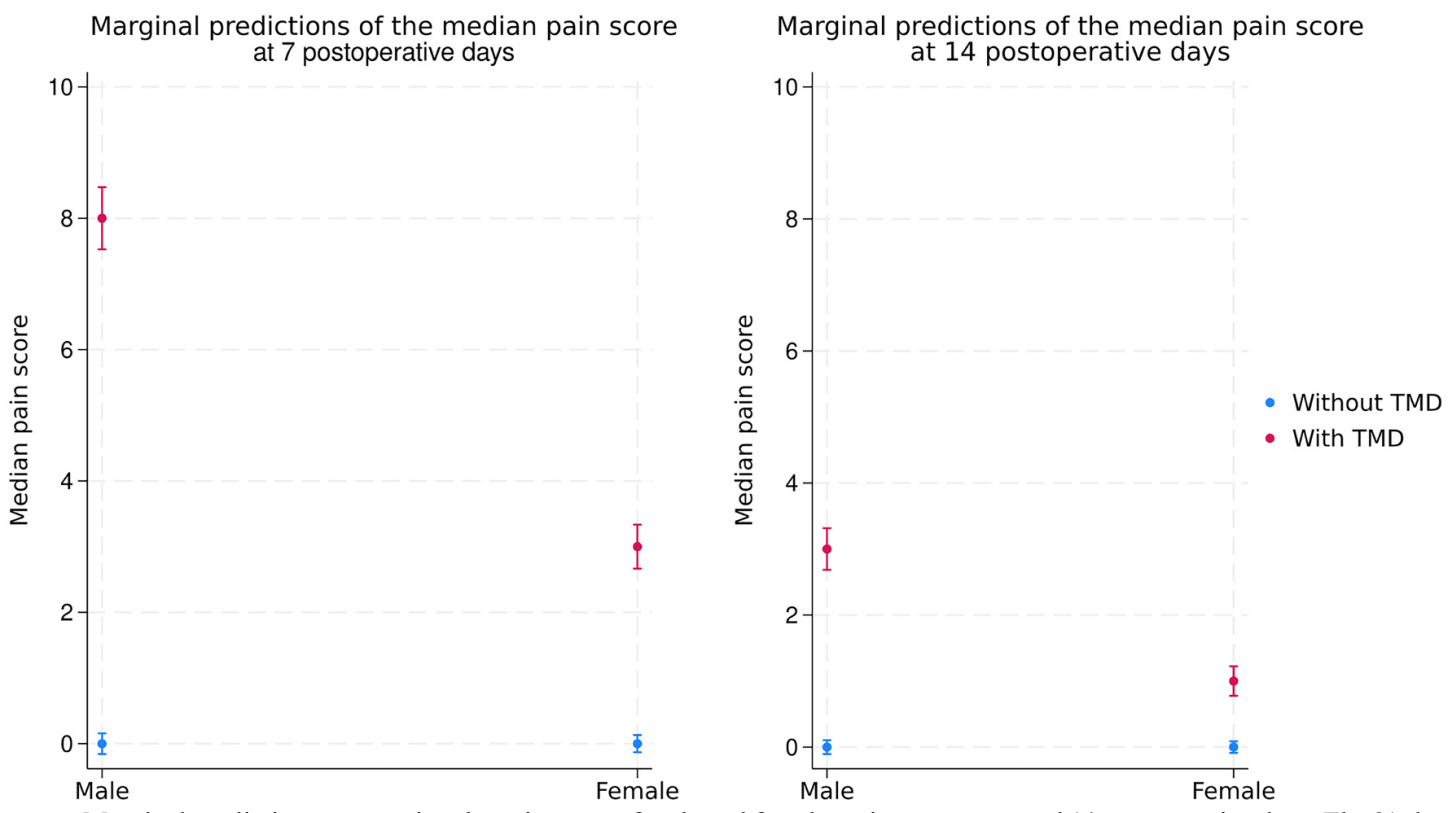
Marginal predictions, comparing the pain score of male and female patients at seven and 14 postoperative days. The 21-day comparison was not included due to the low pain score variability.

Figure 3 analyzed the influence of sex and TMD at seven and 14 postoperative days. Regardless of patient sex, the median pain score was higher in patients with than without TMD. Also, perceived pain scores were higher in male than female patients at seven and 14 days. At seven days, the median score predicted for male patients with TMD was eight, while female patients showed a score of three - a five-point difference (95%CI = 4.4; 5.6), which is considerable because the pain scale has a range of ten points. Similarly, the median pain score of male patients was three at 14 days, while female patients showed a score of one - a two-point difference (95%CI = 1.6; 2.4). All differences between sexes and TMD groups were statistically significant.

The low data variability did not allow testing between sexes and TMD status at 21 days or differences regarding surgery duration (total open mouth time).

## Discussion

This prospective randomized clinical trial assessed pain perception and morbidity after dental implant surgeries in patients with and without TMD. Postoperative pain and morbidity levels were higher in patients with previous TMD, descending over the analyzed days.

Chatzopoulos *et al*. (12) analyzed the relationship between TMD symptoms, bruxism, and dental implant losses, not finding associations between TMD symptoms and implant losses. Conversely, Khan *et al*. (13) presented a more significant relationship between dental and facial trauma - considering adequate proportions - and TMD symptoms that may indirectly accelerate post-traumatic headaches or affect adjacent muscles, joints, and the cervical spine.

Regardless of sex, the median pain score was higher in patients with than without TMD. However, pain scores were higher at seven and 14 days in male than female patients, without considering TMD. The pain regulatory mechanisms behind TMD work differently between sexes (14), even though painful TMD is more frequent in women (15,16).

Our study found twice as many female patients with TMD as male patients. Bueno *et al*. (17) showed that women have double the risk of developing TMD than men. They also emphasize relevant factors recently reported in the literature, such as self-assessed overall health conditions, general disorders, chronic pain, age, study location, ethnicity, and psychosocial and genetic aspects.

Pain decreased over the analyzed time. Mongia *et al*. (18) evaluated three protocols for postoperative pain management in oral surgeries: Group A) Standard analgesic regime with ibuprofen; Group B) A combination of analgesics including paracetamol and diclofenac; and Group C) Adjuvant non-pharmacological interventions using ice pads/cold therapy. The third group demonstrated lower pain intensity scores, lower analgesic intake, and lower incidence of adverse events than the two other groups. However, pain management must adapt to the needs of each patient for an overall pain control improvement.

The group without TMD showed statistical differences of higher pain on the seventh compared to the 14th day due to the high score recorded on the VAS on the first day. Pain analyses might include the pain origin bias, whether caused by the procedure or some exacerbated TMD symptom. Moreover, pharmacological agents are also confounders due to potential interactions, adverse effects, and improved treated conditions.

This study is not free of limitations. The surgical time variation according to professional experience, procedure complexity, and oral limitation measurement only by self-perception implies a careful interpretation of results. The lack of investigations of other factors, such as overall and oral health conditions and the presence or absence of comorbidities or risk factors directly affecting pain perception, are also limitations.

Further randomized clinical trials on oral implant rehabilitation in TMD patients are required to reinforce the findings of this study and provide more precise clinical instructions to prevent and treat this disorder in patients with dental implants. Cone-beam computed tomography combined with mandibular movement tracking software, for instance, presents high accuracy in prosthetic precision, reducing operation time and the number of appointments to produce the prosthesis on the implant (19), thus promoting higher satisfaction during the rehabilitation procedure.

## Conclusions

Patients diagnosed with TMD before the dental implant surgery presented higher pain and morbidity levels on the first 14 postoperative days than patients without TMD. However, pain significantly decreased over time.

## Figures and Tables

**Table 1:** Patient characteristics according to the analyzed groups and variables.

	Total	TMD		p-value
		Without TMD	With TMD	
N	50 (100.0%)	44 (88.0%)	6 (12.0%)	
Sex				
Male	23 (46.0%)	21 (47.7%)	2 (33.3%)	0.674
Female	27 (54.0%)	23 (52.3%)	4 (66.7%)	
Surgery duration				
< 2 hours	15 (30.0%)	12 (27.3%)	3 (50.0%)	0.348
2+ hours	35 (70.0%)	32 (72.7%)	3 (50.0%)	
Pain on day 7^a^	0.0 (0.0; 1.0)	0.0 (0.0; 0.0)	3.5 (3.0; 5.0)	<0.001
Pain on day 14^a^	0.0 (0.0; 0.0)	0.0 (0.0; 0.0)	1.5 (1.0; 3.0)	0.002
Pain on day 21^a^	0.0 (0.0; 0.0)	0.0 (0.0; 0.0)	0.0 (0.0; 0.0)	1.000

a Pain score median and interquartile range; group comparisons using the Kruskal-Wallis test.

## Data Availability

The datasets used and/or analyzed during the current study are available from the corresponding author.
